# Evaluation of microbiological, antioxidant, thermal, rheological and sensory properties of ice cream fermented with kefir culture and flavored with mint (*Menthaspicata* L.)

**DOI:** 10.1002/fsn3.4355

**Published:** 2024-07-21

**Authors:** Feyza Öztürk‐Yalçın, Bayram Ürkek, Mustafa Şengül

**Affiliations:** ^1^ Siran Mustafa Beyaz Vocational School Gumushane University Siran Gumushane Turkey; ^2^ Faculty of Agriculture, Department of Food Engineering Ataturk University Erzurum Turkey

**Keywords:** antioxidant activity, ice cream, kefir culture, lactic acid bacteria, mint flavor, physicochemical properties

## Abstract

Kefir is a healthy fermented dairy product, while ice cream is one of the most consumed dairy products. In this research, ice cream was fermented using a commercial kefir culture and flavored with a mint aroma in different proportions: 0% (KI), 0.2% (KIM2), 0.4% (KIM4), and 0.6% (KIM6). The study investigated the microbiological, thermal, rheological, textural, compositional, and sensory properties as well as antioxidant activity of kefir ice cream samples during 45‐day storage. The lactic bacilli, lactic cocci, and *Leuconostoc* counts of samples were >7 log CFU/g, while the yeast counts were <4 log CFU/g. The microbiological properties (except for yeast counts) of the samples were coherent with legal limits. Antioxidant values (except for samples KIM2 and KIM6) values did not indicate significant differences (*p* < .05). The pH and melting rate values of the samples decreased with the addition of mint flavor, while acidity values increased. The addition of mint aroma caused significant changes in the thermal properties of ice cream. The overrun, a*, WI, and hardness values of the samples decreased based on the mint flavor concentration, whereas the viscosity and rheological increased. Samples KI and KIM2 were scored higher than other samples for all sensory properties. As a result, fermented ice cream flavored (0.2% mint) with mint could be produced as a desirable dairy product with potential functional properties.

## Graphical abstract

This study introduces fermented ice cream production and the effects of using mint aroma. Microbiological, physicochemical, thermal, rheological, and sensory properties as well as antioxidant activity of ice cream samples were investigated.

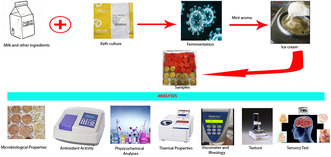

## 1 INTRODUCTION

Ice cream, a frozen dairy product with a wide range of tastes, high commercial potential, and acceptable sensory properties is consumed by people of all ages. Probiotic cultures and healthy ingredients can be used in ice cream formulations. Therefore, ice cream has a potential that can be diversified and improved (Januário et al., 2018). Ice cream is a suitable medium for probiotic bacteria (Guler‐Akin et al., 2016). Many lactic acid bacteria and fungi can be used in probiotic ice cream (Di Criscio et al., 2010). Some researchers produced ice cream samples containing *Lactobacillus acidophilus* and *Bifidobacterium lactis* (in yogurt ice cream) (Ahmad et al., 2020), *Bifidobacterium breve* Bb‐12 and *Lactobacillus plantarum* (Shazly et al., 2022), and *Bifidobacterium longum* subsp. *longum* TISTR (Toommuangpak & Thaiudom, 2024). Kefir contains probiotic bacteria (thermophilic and mesophilic) and yeasts. Kefir has many health‐beneficial effects on human health (Bengi et al., 2023). Kefir could be used as a probiotic microbiota in ice cream production (Al, 2018; Köroğlu, 2015).

Kefir is a fermented dairy product that contains a variety of microorganisms, including lactic acid bacteria, acetic acid bacteria, and yeast. The production of kefir involves using kefir grains and cultures (Gul et al., 2015; Ürkek et al., 2011). Kefir has numerous health benefits, including antimicrobial, antitumor, antioxidant, antimutagenic, cholesterol‐lowering, immune system‐stimulating, and anti‐apoptotic effects (Hertzler & Clancy, 2003). It is considered a probiotic dairy product due to its probiotic bacteria content (Gul et al., 2015). Kefir is a drinkable product with a slightly sour, refreshing taste (Otles & Cagindi, 2003; Yilmaz‐Ersan et al., 2018).

Different ingredients are added to balance the flavor of probiotic ice cream (Hanafi et al., 2022; Shazly et al., 2022). Mint may be used as an aroma compound in the ice cream production. Mint (*Menthaspicata* L.) belongs to the *Labiatae* family, a perennial and aromatic plant (Baliga & Rao, 2010). The essential oil obtained from mint is commonly used as a flavoring agent in tea, ice cream, toothpaste, chewing gum, and confectionery formulations (Asghari et al., 2018; Siano et al., 2005; Verma et al., 2018). Mint chocolate chip ice cream is one of the most popular ice cream flavors. Some of the countries with the highest consumption of mint chocolate chip ice cream are Ecuador, Madagascar, New Zealand, Russia, and Tunisia (Anonymous, 2021).

Studies on the combination of kefir and mint flavor are quite limited. In this study, the probiotic properties of kefir and the refreshing properties of mint flavor were combined in ice cream. The objectives of the research were (a) to determine the effects of mint and storage on microbiological, physicochemical, and sensory properties and antioxidant activity, and (b) to investigate the thermal and rheological properties of mint chocolate chip ice cream.

## 2 MATERIALS AND METHODS

### 2.1 Material

Raw milk was purchased from the Şiran Dairy Products Plant in Gümüşhane, Turkey. The characteristics of the raw milk are shown in Table 1. Kefir starter culture was obtained from CHOOZIT® Kefir DC (LYO 1000 l, Danisco, Germany). Skimmed milk powder was provided by Aynes Dairy Products Co. (Turkey), and mint flavor by Aromsa Co. (İstanbul, Turkey). The stabilizer (salep), emulsifier, butter, and sugar were provided by local markets.

**TABLE 1:** The properties of raw milk.

Parameters	Raw milk
Fat (%)	3.4
Total solids (%)	8.5
Specific gravity (g/mL)	1.0288
Protein (%)	3.1
Lactose (%)	4.6
Ash (%)	0.6
Freezing point°C	−0.539
Conductivity (mS/cm)	5.0

### 2.2 Ice cream production

The ice cream mix recipe was adjusted as milk 72%, sugar 17%, butter 6.25%, emulsifier 0.2%, stabilizer (salep) 0.7%, and skimmed milk powder 3.85%. The mixture was pasteurized at 85°C for 25 s and then cooled to 25°C. The mixture was divided equally into four glass containers (2 kg). Kefir starter culture was added (%2) to each part and kept at 25°C for 24 h for fermentation. The fermented mixtures were kept at +4°C (refrigerator) for one night. The mint flavor was added to the fermented mix before transferring it to the ice cream machine (Sage BCI 600 BSS, Australia). The samples were coded as no mint KI, 0.2% mint KIM2, 0.4% mint KIM4, and 0.6% mint KIM6.

### 2.3 Methods

#### 2.3.1 Microbiological analyses

The lactic bacilli, lactic cocci, *Leuconostoc*, and yeast counts of the samples were determined to assess the microbiological properties of the samples. The analyses were performed using decimal serial dilutions. Lactic bacilli on De Man‐Rogosa‐Sharpe agar (Biolife, Italy) and lactic cocci on M17 agar (Condalab, Madrid, Spain) were determined anaerobically at 30°C for 48–72 h and anaerobically at 30°C for 72 h, respectively. *Leuconostoc* counts were determined on Mayeux, Sandine & Elliker (MSE; Condalab, Madrid, Spain) agar aerobically at 22°C for 120 h (García Fontán et al., 2006). Dichloran‐Rose Bengal Chloramphenicol agar (DRBC, Merck, Germany) for yeast colonies was used after incubation at 25°C for 5–7 days (Jarvis, 1973).

#### 2.3.2 Antioxidant activity

The extract of ice cream samples for DPPH free radical scavenging activity and TPC analysis was obtained with the method with some modifications according to Şengül et al. (2012). Briefly, 25 g samples were weighed and added to 75 mL (90%, v/v) ethanol. The samples were placed on an orbital shaker, shaken at 210 rpm (for 6 h) and filtered through the filter paper.

##### Total phenolic content

Total phenolic content (TPC) values were determined by the colorimetric method described by Gülçin et al. (2002). The prepared extract (10 mL) and 10 mL ethanol (90%, v/v) were placed in an orbital shaker and shaken for 30 min. One mL of the mixture, 46 mL of deionized water, and 1 mL of Folin–Ciocalteu were placed in a glass jar and allowed to stand for 3 min at room temperature. Two percent Na_2_CO_3_ (w/v; Merck, Germany) was added to the mixture and shaken at 210 rpm for 2 h. The absorbance of the prepared mixture was determined using a visible spectrophotometer (DU 730 Beckman Coulter, Inc., Fullerton, CA) at 760 nm against blank samples. The results obtained were expressed as micrograms of gallic acid equivalents per milligram of sample (μg GAE/mg).

##### Scavenging activity on 2,2‐diphenyl‐1‐picryl‐hydrazyl (DPPH) free radicals

0.06 mM DPPH (in ethanol v/v) was prepared. DPPH (2000 μL) and 100, 150, 200, and 250 μL extracts were transferred to flasks. The same amounts of extracts were transferred to another flask and then completed with absolute ethanol. This mixture was used as a blank. All mixtures were vortexed and kept in the dark for 30 min. The absorbance values of the samples were then measured using a visible spectrophotometer (DU 730 Beckman Coulter, Inc., Fullerton, CA) at 517 nm against the blank (Gülçin, 2010). The IC50 values of the samples were calculated with the obtained linear regression using the absorbance values. The IC50 values were expressed as μg/ml.

#### 2.3.3 Thermal measurements

The thermal properties of the ice cream samples were determined using a Differential Scanning Calorimetry (DSC) instrument (EXTAR DSC 7020, Hitachi High‐Tech Corporation, Tokyo, Japan). Weighted (15 g) samples in aluminum pans were placed in the DSC. The instrument was standardized with mercury and indium. The analysis was performed as follows: (a) cooling to −80°C at 10°C/min; (b) heating between −80 and −40°C and tempering at the same temperatures for 30 min to allow maximum ice formation; (c) cooling to −80°C at 10°C/min and isothermal hold for 5 min; and (d) heating between −80 and 20°C at 5°C/min (Blond, 1994).

#### 2.3.4 Proximate analyses

The total solids, ash, fat, protein, pH, and titratable acid contents of the ice cream samples were determined using gravimetric methods (AOAC, 1990). The overrun ratio was measured using the method defined by Akbari et al. (2016). Overrun values were calculated according to Equation (1).

(1) $$\text{Overrun}\;\left(\%\right)=\left[\frac{\left(\text{weight of}\;\mathrm{mix}\right)-\left(\text{weight of}\;\mathrm{ice}\;\text{cream}\right)}{\text{weight of}\;\mathrm{ice}\;\text{cream}}\times 100\right]$$

To determine the first dripping time, 25 g of each sample was weighed and held on a wire mesh at 20°C. The fall time of the first dripping was recorded in seconds (Güven & Karaca, 2002). The amount of melted ice cream samples was determined every 10 min for 70 min. The melting rate was calculated by linear regression and expressed in g/min. A colorimeter (Minolta Colorimeter CR‐400, Osaka, Japan) machine was used to measure L*, a*, b*, the saturation (C*) and Hue angel (*H*°) values. The colorimetric measurements were performed in triplicate and with calibration at *Y* = 92.5, *x* = 0.3060, *y* = 0.3310. The white index (WI) was calculated according to Equation (2), as described by Kurt and Atalar (2018). The equation is as below.

(2) $$\mathrm{WI}=100-\sqrt{{\left(100-L^{*}\right)}^{2}+{\left(a^{*}\right)}^{2}+{\left(b^{*}\right)}^{2}}$$

Viscosity measurements at 20 and 50 rpm were made using a Brookfield Viscometer Model DV‐II (Stoughton, MA, USA). Rheological properties were determined using the power law model after measurements at 5, 10, 20, 50, and 100 rpm. The consistency coefficient (*K*, Pa s^*n*^) and the flow index (*n*) were calculated from the power law model. All viscosity values were determined using spindle no. 6. The power law model was calculated according to Equation (3).

(3) $$\eta ={K\gamma }^{\left(n-1\right)}$$

*η*, apparent viscosity (Pa s); *K*, coefficient consistency (Pa s^*n*^); *γ*, shear rate (s^−1^); *n*, flow behavior index.

#### 2.3.5 Instrumental hardness

A texture analyzer (TA‐XT plus Texture Analyzer Stable Micro Systems Ltd, Godalming, Surrey, UK) was used to measure the hardness of the samples. Hardness values were determined using probe no. P5 at −6°C. The instrument evaluation conditions for pre‐test and test, post‐test speed force, and distance were 2 mm/s, 5 mm/s, 10 g, and 10 mm respectively.

#### 2.3.6 Sensory test

The sensory test of ice cream samples was rated by nine panelists according to the hedonic scale. The panelists were educated about the subject of sensorial test (for 40 h). The ice cream samples were rated from worst 1 to best 9. The panelists were four women and five men aged between 20 and 40. The samples were evaluated in terms of color, gumming structure, flavor, meltdown in the mouth, resistance to melting, and overall acceptability by the panelists (Meilgaard et al., 1999). The required ethical approval for the sensory test was obtained from the Research Ethics Committee of Gümüshane University (approval no. 2021/1 and date 04/02/2021).

### 2.4 Statistical analyses

All data were analyzed using the SPSS17 statistical software package. Significant differences were identified using one‐way ANOVA analysis and Duncan's multiple comparison tests. The study was carried out in two replications. The pH, acidity, first drip time, melting rate, antioxidant activity, microbiological, and sensory analyses were conducted during 45 days of storage (2, 15, 30, and 45 days), while other analyses were carried out only at the beginning of storage.

## 3 RESULTS AND DISCUSSION

### 3.1 Microbiological properties

Lactic bacilli, lactic cocci, *Leuconostoc*, and yeast counts of the ice cream samples are shown in Table 2. Lactic bacilli count of the samples was higher at the end of storage than at the beginning of storage except for sample KIM2, but the change was not statistically significant (*p* > .05). Only on the 15th day of storage, the differences between the samples were statistically significant (*p* < .05). The number of lactic cocci of sample KI increased at the end of storage, whereas sample KIM4 decreased (*p* < .05). There were no significant differences between samples during storage in terms of lactic cocci and *Leuconostoc* counts (*p* > .05). The *Leuconostoc* counts of the samples increased at the 45th day of storage according to the 2nd day of storage, but these changes were not statistically significant (*p* > .05) except for sample KIM4 (*p* > .05). Lactic bacilli, lactic cocci, and *Leuconostoc* counts of all samples were >8 log CFU/g (Table 2).

**TABLE 2:** Microbiological properties of the produced kefir ice creams using the mint aroma (log CFU/g).

Parameters	Storage (days)	KI	KIM2	KIM4	KIM6
Lactic bacilli	2	8.34 ± 0.26A,a	8.44 ± 0.10A,a	8.36 ± 0.07A,a	8.43 ± 0.22A,a
	15	8.29 ± 0.02A,ab	7.70 ± 0.07A,b	8.32 ± 0.07A,a	7.86 ± 0.42A,ab
	30	8.33 ± 0.12A,a	7.96 ± 0.12A,a	8.21 ± 0.24A,a	8.54 ± 0.33A,a
	45	8.56 ± 0.02A,a	7.95 ± 0.54A,a	8.43 ± 0.01A,a	8.51 ± 0.10A,a
Lactic cocci	2	8.68 ± 0.13A,a	8.71 ± 0.08A,a	8.71 ± 0.13A,a	8.68 ± 0.19A,a
	15	8.43 ± 0.00AB,a	8.17 ± 0.98A,a	8.87 ± 0.03A,a	8.26 ± 1.02A,a
	30	8.09 ± 0.20B,a	8.47 ± 0.03A,a	8.88 ± 0.12A,a	8.52 ± 0.61A,a
	45	8.72 ± 0.08A,a	8.01 ± 0.53A,a	8.32 ± 0.14B,a	8.49 ± 0.24A,a
*Leuconostoc*	2	8.35 ± 0.01A,a	8.36 ± 0.12A,a	8.38 ± 0.06C,a	8.47 ± 0.06A,a
	15	8.54 ± 0.53A,a	7.94 ± 1.33A,a	8.95 ± 0.14A,a	8.33 ± 0.90A,a
	30	8.63 ± 0.23A,a	8.32 ± 0.13A,a	8.79 ± 0.14AB,a	8.51 ± 0.69A,a
	45	8.83 ± 0.00A,a	8.63 ± 0.04A,a	8.61 ± 0.05 BC,a	8.60 ± 0.21A,a
Yeast	2	4.01 ± 0.07A,a	3.95 ± 0.10AB,a	3.77 ± 0.08A,a	3.89 ± 0.17AB,a
	15	3.89 ± 0.09AB,a	4.00 ± 0.03A,a	3.99 ± 0.10A,a	4.20 ± 0.51A,a
	30	3.68 ± 0.13B,a	3.96 ± 0.04AB,a	3.97 ± 0.35A,a	3.83 ± 0.19AB,a
	45	3.91 ± 0.04AB,a	3.64 ± 0.21B,a	3.60 ± 0.14A,ab	3.27 ± 0.06B,b

*Note*: Different capital letters (A, B, C, D) indicate statistical significance in the columns, while different small letters (a, b, c, d) indicate in the rows (*p* < .05).

Abbreviations: KI, kefir ice cream without mint aroma; KIM2, kefir ice cream containing 0.2% mint aroma; KIM4, kefir ice cream containing 0.4% mint aroma; KIM6, kefir ice cream containing 0.6% mint aroma.

O'Brien et al. (2016) found that lactic bacilli and lactic cocci counts of traditionally and commercially produced frozen kefir samples decreased at the end of storage (day 30). They determined lactic bacilli counts to be 7.24 log CFU/mL (traditional) and 6.33 log CFU/mL (commercial), and lactic cocci counts to be 6.24 log CFU/mL (traditional) and 5.44 log CFU/mL (commercial) at the end of storage. Sarica and Coşkun (2022) investigated some properties of frozen kefir samples prepared from cow's and goat's milk lactic bacilli, lactic cocci, and *Leuconostoc* counts for 45 days. They found a decrease in the number of lactic bacilli, lactic cocci, and *Leuconostoc* counts at the end of storage. The results obtained in the present study were not consistent with those reported by O'Brien et al. (2016) and Sarica and Coşkun (2022) and were higher than their results at the end of storage.

Akca and Akpinar (2021) investigated some properties of probiotic ice cream (*Lactobacillus rhamnosus* and *Bifidobacterium animalis* subsp. *lactis* BB‐12) containing sesame and pomegranate powder and oil at different ratios. They reported that the bacteria viability of ice creams did not change significantly depending on sesame and pomegranate powder and oil. Shazly et al. (2022) researched the microbiological and physicochemical properties of probiotic (*Lactobacillus plantarum* and *Bifidobacterium breve*) ice creams added to the concentrated coffee extract. They did not find a significant difference in microbiota counts of probiotic ice cream containing concentrated coffee extract. In the current study of the microbiota, results were similar as reported by Akca and Akpinar (2021) and reported by Shazly et al. (2022).

The yeast counts of the samples ranged from 3.77 log CFU/g to 4.01 log CFU/g on the 2nd day of storage, while at the end of storage they ranged from 3.27 log CFU/g to 3.91 log CFU/g (Table 2). All samples had lower yeast counts at the end of storage than on the 2nd day of storage (*p* < .05), except KIM4. At the end of storage, sample KIM6 had the lowest yeast count (3.27 log CFU/g). There were no noticeable differences observed between the samples during the remaining storage days, as per the statistical analysis (*p* > .05). O'Brien et al. (2016) determined yeast counts between 8.83 log CFU/g (day 1) and 6.82 log CFU/g (day 30) in traditional samples, and 7.20 log CFU/g (day 1) and 4.38 log CFU/g (day 30) in commercial samples. Sarica and Coşkun (2022) found a decrease during storage and, the yeast values were less than 2 log CFU/mL at the end of storage. Similarly, Al (2018), and Köroğlu (2015) found a decreasing yeast count in kefir ice cream (KIC) samples at the end of storage. The results of the present study were lower than those reported by O'Brien et al. (2016), while they were higher than those reported by Al (2018), Köroğlu (2015), and Sarica and Coşkun (2022).

According to legal requirements, the kefir microbiota must contain at least 7 log CFU/g of specific microorganisms and 4 log CFU/g of yeast (Codex Alimentarius Commission, 2022; Turkish Food Codex, 2009). All KIC samples complied with the legal requirements for lactic bacilli, lactic cocci, and *Leuconostoc* counts, but not for yeast counts.

### 3.2 Antioxidant activity

The DPPH radical scavenging activities of the samples were determined as IC50 values. There is an inverse relationship between DPPH radical scavenging activities and IC50 values. During the whole storage period, there was no significant difference in IC50 values between samples (*p* > .05; Table 3). At the end of storage, IC50 values ranged from 588.77 to 697 μg/mg for KI and KIM2, respectively.

**TABLE 3:** Antioxidant capacity of the produced kefir ice creams using the mint aroma.

Parameters	Storage (days)	KI	KIM2	KIM4	KIM6
DPPH (IC50, μg/mg)	2	736.22 ± 72.03A,a	740.07 ± 146.47A,a	698.94 ± 112.31A,a	594.75 ± 239.20A,a
	15	565.81 ± 72.28A,a	547.98 ± 82.96A,a	544.07 ± 61.21A,a	515.59 ± 21.64A,a
	30	746.40 ± 99.77A,a	651.40 ± 3.49A,a	595.98 ± 29.01A,a	637.67 ± 129.28A,a
	45	588.77 ± 2.44A,a	697.55 ± 68.87A,a	644.76 ± 90.75A,a	629.65 ± 28.85A,a
TPC (μg GAE/mg)	2	75.54 ± 6.90A,a	71.88 ± 8.62B,a	71.88 ± 5.17A,a	88.95 ± 1.72AB,a
	15	76.76 ± 18.96A,a	67.00 ± 5.17B,a	76.76 ± 15.51A,a	86.51 ± 5.17AB,a
	30	95.04 ± 6.90A,a	87.73 ± 3.45A,a	95.04 ± 10.34A,a	103.57 ± 5.17A,a
	45	86.51 ± 1.72A,a	74.32 ± 12.07AB,a	88.95 ± 5.17A,a	77.98 ± 10.34B,a

*Note*: Different capital letters (A, B, C, D) indicate statistical significance in the columns, while different small letters (a, b, c, d) indicate in the rows (*p* < .05).

Abbreviations: DPPH, 2,2‐diphenyl‐1‐picryl‐hydrazyl; KI, kefir ice cream without mint aroma; KIM2, kefir ice cream containing 0.2% mint aroma; KIM4, kefir ice cream containing 0.4% mint aroma; KIM6, kefir ice cream containing 0.6% mint aroma; TPC, total phenolic components.

The TPC values of the samples are shown in Table 3. Sample KIM2 had higher TPC values at the end of storage than at the beginning of storage, while sample KIM4 had a lower value at the end of storage (*p* < .05). Sample KIM6 had the highest TPC values except for the 45th day of storage. However, these differences were not statistically significant (*p* > .05).

Akca and Akpinar (2021) found that the TPC and antioxidant capacity of ice cream containing sesame, pomegranate, and grape seed oils were between 45.50 and 70.63 mg GAE/g and 23.31%–37.43%, respectively. Amin et al. (2023) determined TPC and DPPH values of ice cream made with chia seed oil to be 0.11–5.19 mg GAE/mL and 5.62%–51.18%, respectively. They observed that TPC and DPPH values increased with increasing chia seed oil concentration.

The IC50 and TPC values of the mint flavor were 195.28 μg/mg and 392.46 μg GAE/mg, respectively. The mint flavor did not affect the IC50 and TPC values of the ice cream samples. This situation may have been caused by the lower amount of mint flavoring used. Additionally used aroma type would have had effects on antioxidant activity.

### 3.3 pH, acidity, first dripping, and melting rate properties

KIC samples had the lowest pH values at the end of storage (Table 4). However, these changes were statistically significant only for samples KI (pH 4.67) and KIM4 (pH 4.65) (*p* < .045). Throughout the entire storage period, the KIM2 sample exhibited the highest pH values. The acidity values of the KIC samples showed no significant differences during the whole storage, except for sample KIM4 (*p* > .05). The acidity of sample KIM6 was lower than the other samples on the 2nd and 15th day of storage (*p* > .05). At the end of storage, the acidity values were between 0.68% and 0.82% (Table 4) and the differences among the samples were not significant (*p* > .05). Köroğlu (2015) found the pH and acidity values of KIC samples to be between 5.85%–6.13% and 0.25%–0.26%, respectively, during 30 days of storage. Al (2018) found that pH and acidity values of KIC samples changed between 5.51%–6.38% and 0.18%–036% during 90‐day storage, respectively. The pH values obtained in the present study were lower than those reported by Al (2018) and Köroğlu (2015), while the acidity values were higher. Macit et al. (2017) investigated some characteristics of ice cream with different spice oils. Çam et al. (2013) investigated the properties of ice cream containing pomegranate phenols and oil. Macit et al. (2017) found that the pH and acidity values of ice creams did not show significant changes, while Çam et al. (2013) reported that the pH and acidity were significantly different. It has been speculated that the type and amount of added ingredients may have been affected by pH and acidity.

**TABLE 4:** pH, acidity, first dripping, and melting rate values of the produced kefir ice creams using the mint aroma.

Parameters	Storage (days)	KI	KIM2	KIM4	KIM6
pH	2	4.85 ± 0.00B,b	4.97 ± 0.00A,a	4.73 ± 0.00B,d	4.77 ± 0.03A,c
	15	4.87 ± 0.01AB,b	4.97 ± 0.01A,a	4.74 ± 0.02B,c	4.77 ± 0.01A,c
	30	4.89 ± 0.01A,b	4.98 ± 0.04A,a	4.81 ± 0.02A,bc	4.79 ± 0.05A,c
	45	4.67 ± 0.02C,b	4.92 ± 0.08A,a	4.65 ± 0.01C,b	4.72 ± 0.01A,b
Acidity (% lactic acid)	2	0.77 ± 0.01A,b	0.77 ± 0.03A,b	0.77 ± 0.03AB,b	0.86 ± 0.00A,a
	15	0.78 ± 0.03A,ab	0.69 ± 0.08A,b	0.85 ± 0.07A,ab	0.90 ± 0.06A,a
	30	0.85 ± 0.03A,ab	0.79 ± 0.04A,b	0.90 ± 0.00A,a	0.85 ± 0.01A,ab
	45	0.82 ± 0.09A,a	0.68 ± 0.04A,a	0.70 ± 0.06B,a	0.82 ± 0.07A,a
First dripping time (s)	2	2580.00 ± 0.00A,a	2850.00 ± 381.84A,a	3000.00 ± 169.71A,a	2640.00 ± 84.85A,a
	15	2970.00 ± 127.28A,a	2490.00 ± 42.43A,b	2880.00 ± 169.71A,a	2910.00 ± 42.43A,a
	30	3030.00 ± 212.13A,a	2610.00 ± 466.69A,a	2700.00 ± 84.85A,a	3150.00 ± 381.84A,a
	45	2850.00 ± 212.13A,a	2610.00 ± 721.25A,a	2550.00 ± 466.69A,a	2580.00 ± 254.56A,a
Melting rate (g/min)	2	0.59 ± 0.00B,c	0.86 ± 0.03A,a	0.79 ± 0.02A,b	0.87 ± 0.01A,a
	15	0.62 ± 0.05B,b	0.85 ± 0.03A,a	0.80 ± 0.04A,a	0.81 ± 0.01A,a
	30	0.80 ± 0.06A,a	0.74 ± 0.01B,ab	0.82 ± 0.03A,a	0.68 ± 0.03B,b
	45	0.79 ± 0.02A,a	0.63 ± 0.01C,b	0.66 ± 0.02B,b	0.65 ± 0.04B,b

*Note*: Different capital letters (A, B, C, D) indicate statistical significance in the columns, while different small letters (a, b, c, d) indicate in the rows (*p* < .05).

Abbreviations: KI, kefir ice cream without mint aroma; KIM2, kefir ice cream containing 0.2% mint aroma; KIM4, kefir ice cream containing 0.4% mint aroma; KIM6, kefir ice cream containing 0.6% mint aroma.

The first dripping times and melting rates of the KIC samples are presented in Table 4. Lactic acid bacteria are responsible for the reduction of pH and increase in acidity through fermentation (Ranadheera et al., 2012; Sah et al., 2016). The lactic acid bacteria count increased at the end of storage (Table 2). The differences in lactic acid bacteria counts may have caused the changes in pH and acidity. The differences in first dripping times were not statistically significant among the KIC samples (except at 15 days) and during storage (*p* > .05). The melting rates of the KIC samples containing mint flavor decreased significantly at the end of storage, while those of the control sample increased (*p* < .05). Kanca et al. (2023) investigated some properties of ice cream samples to which kefir was added at different rates. They found that the first dripping times were between 1920 s and 2400 s, and melting rates of the samples changed from 0.66 to 0.80 g/min. The first dripping values reported by Kanca et al. (2023) were lower than ours and the melting values were similar. But the results determined herein were in disagreement with Güven et al. (2018), who found that the first dripping and complete melting times were higher than other samples. Güven et al. (2018) studied characteristics of ice cream added hazelnut and olive oil. The differences between the previous studies and the present study may be due to the difference in the proportion of fat used in the studies. On the other hand, Macit et al. (2017) determined no effect of spice oil addition on ice cream melting.

The low pH values have an important effect on the melting resistance of ice cream. Research has shown that the structure of proteins is affected by low pH values, which results in high melting resistance (Favaro‐Trindade et al., 2007).

### 3.4 Thermal properties

The midpoint and offset temperatures of glass transition (*T*_*g*_') decreased as a function of mint flavor concentration, while the onset temperatures showed no significant (*p* > .05) changes (Table 5). The onset and midpoint temperatures of melting (*T*_*m*_′) were highest for sample KI, while the offset temperatures did not change significantly among the samples (*p* > .05). Similarly, Ürkek (2021) determined glass transition and melting temperatures of ice cream samples containing a mixture of chia seed powder and salep between (−55.56°C)–(−37.77°C) and (−36.17)–(−26.33°C), respectively. Ertugay et al. (2020) reported similar results when they investigated some properties of ice cream with added Kavılca fiber.

**TABLE 5:** Thermal properties of the produced kefir ice creams using the mint aroma.

Parameters	KI	KIM2	KIM4	KIM6
*T*_*g*_ *' (glass transition temperature)*				
Onset,°C	−53.96 ± 3.12a	−52.63 ± 0.14a	−54.93 ± 0.45a	−52.74 ± 1.21a
Midpoint,°C	−47.36 ± 0.22c	−47.75 ± 0.02b	−48.73 ± 0.01a	−48.84 ± 0.16a
Offset,°C	−41.33 ± 1.99b	−42.13 ± 0.64ab	−44.94 ± 0.17a	−43.44 ± 0.98ab
*T*_*m*_ *ʹ (melting temperature)*				
Onset,°C	−35.12 ± 1.01b	−37.78 ± 0.35a	−38.06 ± 0.48a	−38.89 ± 1.17a
Midpoint,°C	−32.02 ± 0.02b	−32.61 ± 0.97ab	−33.54 ± 0.07a	−33.36 ± 0.34ab
Offset,°C	−29.71 ± 0.04a	−28.79 ± 0.28a	−29.59 ± 0.58a	−28.58 ± 2.19a
Freezing point temperature, *T*_*f*_ (°C)	−14.10 ± 0.08a	−15.37 ± 0.96a	−14.98 ± 1.28a	−13.77 ± 1.99a
Heat of ice freezing, Δ*H* (J/g)	171.50 ± 34.65b	111.50 ± 4.95a	96.00 ± 5.66a	133.00 ± 4.24ab
Melting point temperature, *T*_*m*_ (°C)	3.07 ± 0.16b	1.30 ± 0.18a	2.44 ± 0.73ab	1.30 ± 0.60a
Heat of ice melting, Δ*H* (J/g)	190.00 ± 56.57a	134.50 ± 6.36a	147.50 ± 3.54a	142.00 ± 4.24a

*Note*: Different small letters (a, b, c, d) indicate statistical significance in the rows (*p* < .05).

Abbreviations: KI, kefir ice cream without mint aroma; KIM2, kefir ice cream containing 0.2% mint aroma; KIM4, kefir ice cream containing 0.4% mint aroma; KIM6, kefir ice cream containing 0.6% mint aroma.

The freezing point (*T*_*f*_) and melting point (*T*_*m*_) temperatures were determined between (−13.77°C)–(−15.37°C) and 1.30–3.07°C, respectively (Table 5). Ice freezing values were lower in the mint‐flavored KIC samples than in the control sample. The heat of melting values showed irregular changes and these changes were not significant (*p* > .05). Similar results were reported by Ertugay et al. (2020) and Kavaz Yuksel (2015) who studied some characteristics of ice cream containing sloe berries. Atik et al. (2021) found that thermal values increased in ice cream samples containing chia seed oil. It was found that many factors such as water holding capacity, serum phase ratio, soluble matter rate, freezing ratio, and protein interactions on *T*_*g*_', *T*_*m*_′, *T*_*f*_, and *T*_*m*_ values of ice cream (Soukoulis et al., 2009). Changes in pH and acidity affected protein interactions (Kneifel et al., 1991; Soukoulis & Tzia, 2008). The difference in thermal properties may have been caused by changes in pH and acidity due to microbial activity.

### 3.5 Proximate analysis

There were slight differences in the total solids, ash, fat, and protein values of the KIC samples (Table 6), but these were not significant (*p* > .05). Similarly, Çam et al. (2013), Kanca et al. (2023), Macit et al. (2017), and Nadeem et al. (2010) found no statistical significance in the total solids, ash, fat, and protein values of ice cream.

**TABLE 6:** Proximate composition of the produced kefir ice creams using the mint aroma.

Parameters	KI	KIM2	KIM4	KIM6
Total solids (%)	36.81 ± 0.23a	37.01 ± 0.35a	37.01 ± 0.20a	37.23 ± 0.11a
Ash (%)	0.81 ± 0.00a	0.79 ± 0.03a	0.78 ± 0.00a	0.78 ± 0.01a
Fat (%)	10.00 ± 0.28a	10.30 ± 0.42a	10.10 ± 0.42a	10.90 ± 0.42a
Protein (%)	3.77 ± 0.26a	3.63 ± 0.04a	3.75 ± 0.11a	3.66 ± 0.45a
Overrun (%)	40.26 ± 0.95a	35.15 ± 0.76b	32.50 ± 0.77c	32.44 ± 0.95c
Viscosity at 20 rpm (cP)	12807.38 ± 1709.16a	13203.45 ± 2511.01a	14488.44 ± 729.54a	14554.92 ± 1773.43a
Viscosity at 50 rpm (cP)	6630.08 ± 721.37ab	6266.83 ± 219.44b	7614.35 ± 358.45a	7638.51 ± 244.46a
Consistency coefficient (*K*; Pa s^*n*^)	60.60 ± 11.15ab	54.46 ± 12.20b	57.29 ± 0.99ab	78.30 ± 1.00a
Flow behavior index (*n*)	0.41 ± 0.11a	0.48 ± 0.04a	0.48 ± 0.00a	0.40 ± 0.01a
*R* ^2^	.98 ± .01	.92 ± .04	.94 ± .01	.99 ± .01
L*	97.04 ± 0.38a	96.46 ± 1.44a	97.76 ± 0.45a	97.31 ± 0.09a
a*	−0.91 ± 0.30a	−1.47 ± 0.63ab	−2.13 ± 0.00b	−2.15 ± 0.01b
b*	2.69 ± 0.08a	3.38 ± 1.93a	5.06 ± 0.33a	4.99 ± 0.27a
C*	2.84 ± 0.18a	3.68 ± 2.02a	5.49 ± 0.30a	5.43 ± 0.25a
*H°*	108.40 ± 5.37a	114.40 ± 3.54a	112.80 ± 01.27a	113.20 ± 0.99a
WI	95.90 ± 0.39a	94.61 ± 0.11b	94.06 ± 0.11b	93.94 ± 0.26b
Hardness (*N*)	106.19 ± 2.31a	102.36 ± 7.91a	69.23 ± 6.57b	42.19 ± 2.11c

*Note*: Different small letters (a, b, c, d) indicate statistical significance in the rows (*p* < .05).

Abbreviations: KI, kefir ice cream without mint aroma; KIM2, kefir ice cream containing 0.2% mint aroma; KIM4, kefir ice cream containing 0.4% mint aroma; KIM6, kefir ice cream containing 0.6% mint aroma.

The overrun values of the samples decreased in the mint‐flavored KIC samples, and the control sample had the highest overrun value (40.26%). Kanca et al. (2023) found the lowest overrun value in the control sample. Salem et al. (2005) produced ice cream with *Lb. acidophilus*, *B. bifidum*, *Lb. reuteri*, *Lb. gasseri*, and *Lb. rhamnosus*. They obtained the highest overrun value in the control sample. The results of the present study are in agreement with those reported by Salem et al. (2005) but were not in harmony with those reported by Kanca et al. (2023). Many factors affect the overrun values of ice cream, such as the protein structure, acidity, and freezing point. Changes in the volume increase of ice creams containing microorganisms may be due to the use of different levels of acidity and cultures, which affect the freezing point and the structure of the protein (Salem et al., 2005). Fat globules, protein/emulsifier, and production process affect overrun (Kurultay et al., 2010; Ürkek, 2021). Nadeem et al. (2010) found a decrease in overrun values of ice creams containing rape seed oil (1%, 2% and 3%). Similarly, Nazarewicz et al. (2022) reported that the overrun values decreased while the content of tomato seed oil ratio of the ice cream increased. They have been stated that the vegetable oil ratio affects overrun values (Nadeem et al., 2010; Nazarewicz et al., 2022). These results were similar to the present research. As stated by Macit et al. (2017), the overrun values of ice creams containing different spice oils did not change significantly.

The viscosity values at 20 and 50 rpm of the KI sample were lower than those of the mint‐flavored samples (Table 6). However, this difference was significant only at 50 rpm (*p* < .05). Salem et al. (2005) and Köroğlu (2015) reported that the control samples had lower viscosity than samples containing microorganisms. Nadeem et al. (2010) detected decreasing viscosity values in ice creams with added rape seed oil. Shazly et al. (2022) found that the viscosity values of probiotic ice cream increased based on increments of concentrated coffee extract. The results obtained in this research were similar to those reported by Salem et al. (2005), Köroğlu (2015), and Shazly et al. (2022), while they were in disagreement with Nadeem et al. (2010).

The *K* values of the KIC samples decreased with the addition of 0.2% mint flavor and increased in the other samples (*p* < .05). Kanca et al. (2023) found that the *K* values of ice cream samples containing kefir changed significantly. The results reported by Kanca et al. (2023) were not coherent with the present study. All samples had *n* values between 0.40 and 0.48, and pseudoplastic behavior (Table 6). Many researchers found the flow behavior of ice cream samples as pseudoplastic behavior (Aloğlu et al., 2018; Kanca et al., 2023; Ürkek et al., 2022).

It has been reported that an increase in the water‐holding capacity of milk proteins has a positive effect on viscosity, overrun, and melting. The water‐holding capacity of milk proteins increases as the pH decreases toward the isoelectric point (Kneifel et al., 1991). In addition, exopolysaccharides affect the viscosity and rheological properties of products, and kefir cultures could be produced with exopolysaccharides (Yang et al., 2022). Differences in viscosity and *K* values may have been caused by pH and microbial content.

The colorimetric parameters of the KIC samples did not show any significant changes in terms of L*, b*, C*, and *H*° (*p* > .05; Table 6). Sample KI had the highest a* (−0.91) and WI (95.90) values. The L* values were ranged from 0.92 to 0.99, whereas the WI values varied from 93.94 to 95.90 (Table 6). The L* values did not indicate change significantly among samples, while WI values were higher in the samples containing mint aroma than the control sample (*p* < .05). Öztürk et al. (2018) stated that the color parameters of ice cream samples containing *Lactobacillus casei* 431 and white‐dark myrtus fruit were not affected by the addition of *Lactobacillus casei* 431. Similar results were reported by Güven et al. (2018) who studied some properties of ice creams and added hazelnut and olive oil. There was slight or no difference in the color parameters. This situation might be caused by the use of very low aroma concentration.

### 3.6 Instrumental hardness

As can be seen from Table 6, the hardness values of the KIC samples decreased with the concentration of mint flavor concentration. The hardness values of samples KI and KIM2 were statistically similar (*p* > .05), while the results of these samples were higher than those of the other ice cream samples (*p* < .05). Kanca et al. (2023) found no significant difference between ice cream samples in terms of hardness values. These results are not consistent with those reported by Acu et al. (2021) and Kanca et al. (2023) who investigated some properties of ice cream samples with probiotic starter culture and raspberry added.

There is an inverse relationship between viscosity and hardness (Kurt & Atalar, 2018). The hardness values are affected by the ice crystal size, ice phase, and water‐holding capacity (Muse & Hartel, 2004; Ürkek, 2021). As shown in Table 6, the viscosity values increased while the hardness values decreased. On the other hand, the overrun values decreased while the hardness values decreased (Table 6). Toommuangpak and Thaiudom (2024) investigated the effects on some properties of yogurt ice cream produced using *Bifidobacterium longum* subsp. *longum* TISTR 2195 containing galacto‐oligosaccharide and fructo‐oligosaccharide. They found that overrun values did not indicated a significant change, while hardness values indicated significant differences. Atallah et al. (2022), who studied the properties of ice cream made from buffalo milk, reported that the overrun values decreased, as did the hardness. Nadeem et al. (2010) determined some characteristics of ice cream containing rape seed oil at different ratios (1%, 2% and 3%). Nadeem et al. (2010) reported that overrun values of ice creams containing oil were decreased, whereas the hardness values increased.

The obtained results in the research were similar to the previously reported studies by some researchers (Toommuangpak & Thaiudom, 2024), while they were in disagreement with some researchers (Nadeem et al., 2010; Şentürk et al., 2024; Ürkek, 2021). This situation could have been caused by vegetable fat content, protein structure, and microbial fermentation (Aboulfazli et al., 2015; Pimentel et al., 2022). So, the overrun and hardness properties could have been affected by many factor. On the other hand, using the high oil ratios in previous studies could be the reason for differences compared to the present study.

### 3.7 Sensory test

**FIGURE 1:**
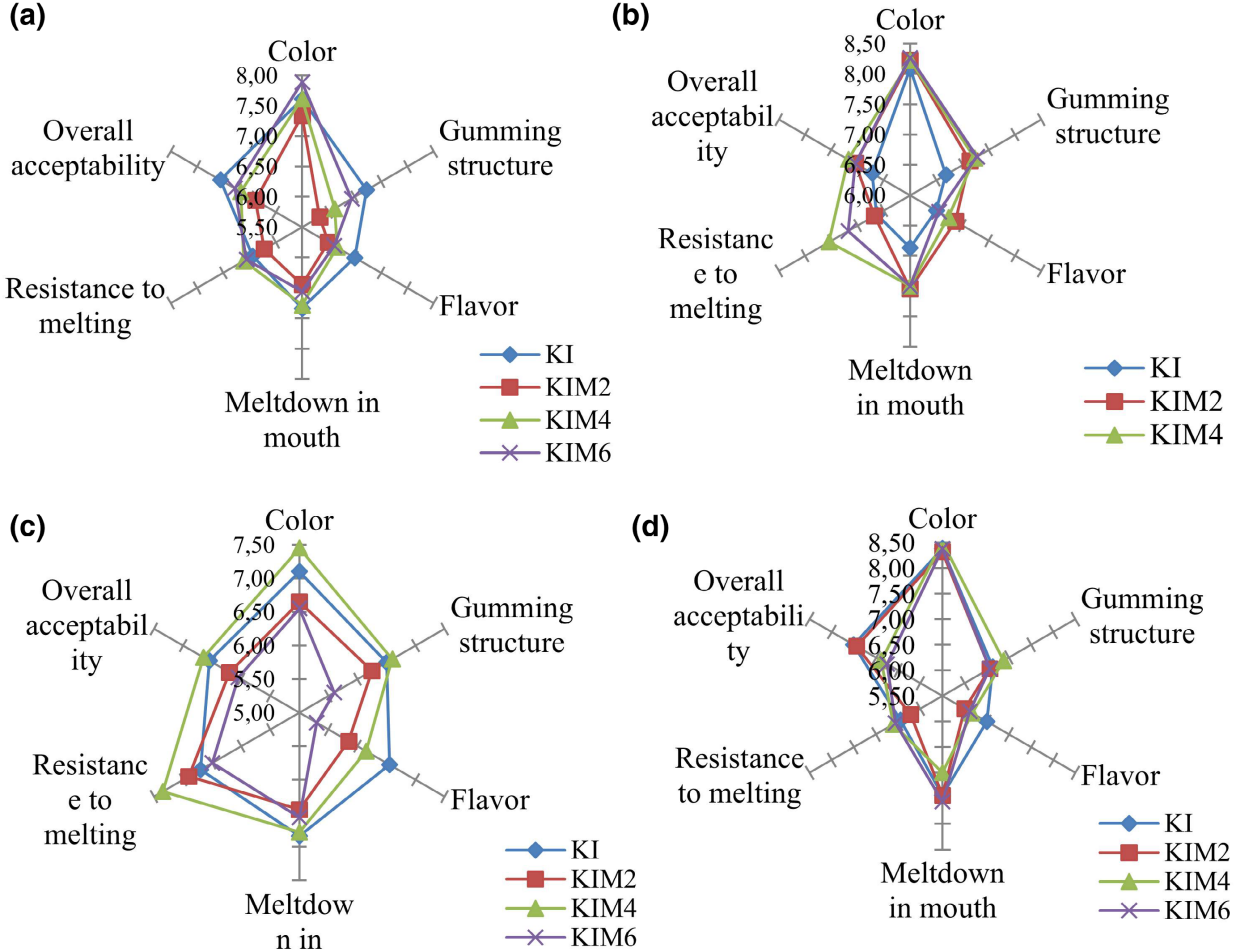
Sensory scores of kefir ice cream samples at the storage of 2nd (a), 15th (b), 30th (c), and 45th (d) days (KI, Kefir ice cream without mint aroma; KIM2, Kefir ice cream containing 0.2% mint aroma; KIM4, Kefir ice cream containing 0.4% mint aroma; KIM6, Kefir ice cream containing 0.6% mint aroma).

The sensory test scores of the KIC samples are shown in Figure 1. All sensory test scores of sample KI were generally the highest during 45 days. The flavor scores of sample KIM6 were the lowest at the end of storage. The overall acceptability of the KIC samples ranged from 6.39 to 7.51, while the flavor scores varied from 5.30 to 7.19. The melting in mouth and resistance to melting scores of the KIC samples showed no significant change between samples and during storage (*p* > .05). Sample KIM2 did have the highest scores than sample KI in terms of flavor and overall acceptability. Color, taste, melting in mouth, and overall acceptability scores of sample KIM2 were higher at the end of storage than at the beginning of storage (*p* < .05). Kanca et al. (2023) found that sensory scores of ice creams with high kefir content were usually lower than other samples. Salem et al. (2005) reported that the total sensory score of the control sample was higher than other samples containing probiotic bacteria.

## 4 CONCLUSION

This study found that the use of mint flavor only had a significant effect on acidity, pH, melting rate (*p* < .01), and WI values (*p* < .05), while storage affected acidity, melting rate, yeast (*p* < .01), lactic bacilli, and pH (*p* < .05). Lactobacilli, lactococci, and *Leuconostoc* counts of all samples conformed to legal requirements (>7 log CFU/g), whereas yeast counts were not in harmony with legal rules (>4 log CFU/g). The highest sensory score was recorded in the KI sample, followed by the sample with 0.2% mint flavor. It could be suggested that kefir cultures with 0.2% mint flavor can be used for ice cream production. Because specific microorganism counts (lactobacilli, lactococci, and *Leuconostoc*) of sample KIM2 were coherent with legal rules, and sensory scores were higher among the samples containing mint flavor. In the present study, the probiotic properties of kefir and the refreshing effect of mint aroma were combined in the ice cream. Thus, a novel ice cream could be produced and contribute to the dairy industry. Future research could investigate the use of different flavoring compounds in kefir ice cream and their effect on the complex structure of the ice cream.

## AUTHOR CONTRIBUTIONS

**Feyza Öztürk‐Yalçın:** Data curation (equal); formal analysis (equal); investigation (equal). **Bayram Ürkek:** Data curation (equal); formal analysis (equal); investigation (equal); methodology (equal); project administration (lead); software (equal); supervision (equal); writing – original draft (equal); writing – review and editing (equal). **Mustafa SENGUL:** Conceptualization (equal); formal analysis (equal); writing – original draft (equal); writing – review and editing (equal).

## CONFLICT OF INTEREST STATEMENT

The authors declare that they have no conflict of interest financial interests or personal.

## Data Availability

The datasets generated during and/or analyzed during the current study are available from the corresponding author upon reasonable request.
